# Cancer Cachexia: Muscle Physiology and Exercise Training

**DOI:** 10.3390/cancers4041247

**Published:** 2012-11-29

**Authors:** Claudio L. Battaglini, Anthony C. Hackney, Matthew L. Goodwin

**Affiliations:** 1 Department of Exercise & Sports Science, University of North Carolina at Chapel Hill, Chapel Hill, NC 27599, USA; E-Mail: claudio@e mail.unc.edu; 2 Department of Nutrition, Gillings School of Global Public Health, University of North Carolina at Chapel Hill, Chapel Hill, NC 27599, USA; 3 Weill Cornell Medical College, New York, NY 10021, USA; E-Mail: mlg2008@med.cornell.edu

**Keywords:** cachectic syndrome, sarcopenia, physical exercise training, muscle accretion

## Abstract

Cachexia in cancer patients is a condition marked by severe tissue wasting and a myriad of quality of life and health consequences. Cachexia is also directly linked to the issues of morbidity and survivability in cancer patients. Therapeutic means of mitigating cachexia and its effects are thus critical in cancer patient treatment. We present a discussion on the use of physical exercise activities in the context of such treatment as a means to disruption the tissue wasting effects (*i.e.*, muscle tissue losses via anorexigenic pro-inflammatory cytokines) of cachexia. In addition we propose a theoretical model (Exercise Anti-Cachectic Hypothetical—“EACH” model) as to how exercise training may promote a disruption in the cycle of events leading to advancing cachexia and in turn promote an enhanced functionality and thus improved quality of life in cancer patients.

## 1. Introduction and Discussion

The cachexia condition accompanies many forms of cancer. This condition is multidimensional and highly complex, and has not been precisely understood or well-defined by the scientific community. To this end, an international panel of leading scientists and physicians recently defined cancer cachexia as “a multifactorial syndrome defined by an ongoing loss of skeletal muscle mass (with or without loss of fat mass) that cannot be fully reversed by conventional nutritional support and leads to progressive functional impairment” [1]. The condition is characterized not only by marked weight loss with muscle and potential adipose tissue wasting, but also asthenia, anorexia, anemia, disturbances in energy balance, and changes in carbohydrate, lipid, and protein metabolism [2,3,4,5,6]. These effects promote a severe series of metabolic disturbances due to the starvation-like state, in particular muscular protein tissue degradation—that is, a loss of muscle mass (*i.e.*, sometimes referred to as sarcopenia). These undesirable effects have been associated with increased morbidity and significant reductions in the quality of life of the cancer patient [3]. Regrettably, the cachectic condition occurs in a majority of cancer patients before their death. Furthermore, it has been reported that cachexia alone is thought to be responsible for approximately 25% of all cancer patients’ deaths [3,4].

Recently in the *New England Journal of Medicine*, the eminent cachexia researcher, Dr. K.C. Fearon commented in a “Clinical Implications” article that in cancer patients, the shortest survival time is usually observed in those experiencing cachexia or a sarcopenia-like condition. Dr. Fearon went on to indicate that research which has examined “the manipulation of the integrative physiology of muscle and adipose tissues for therapeutic gain” in such patients is extremely scant in nature and lacks empirical evidence relative to efficacious treatment [7]. Thus, he concluded there is a need for further research in this area [7].

Our research team for a number of years has taken on this integrative physiology approach in an attempt to examine the influence of exercise training on alleviating the cachectic effects in breast cancer and leukemia patients [8,9]. Our intent has been to demonstrate the positive influence of physical exercise on protein synthesis in skeletal muscle via an up-regulation of anti-inflammatory cytokines and acceleration of myoplasticity events (*i.e.*, protein synthesis) within the skeletal muscle. The results of our preliminary investigations thus far have demonstrated that cancer patients are able to engage in regular exercise training and that physical exercise activities have the potential to shift the balance of protein turnover towards an anabolic *vs*. catabolic state. That is, allowing the promotion of muscle mass retention or even the hypertrophy of various muscle tissues. Our work is not alone in this respect and subsequent investigations by others are also supportive of these outcomes [10,11].

**Figure 1:**
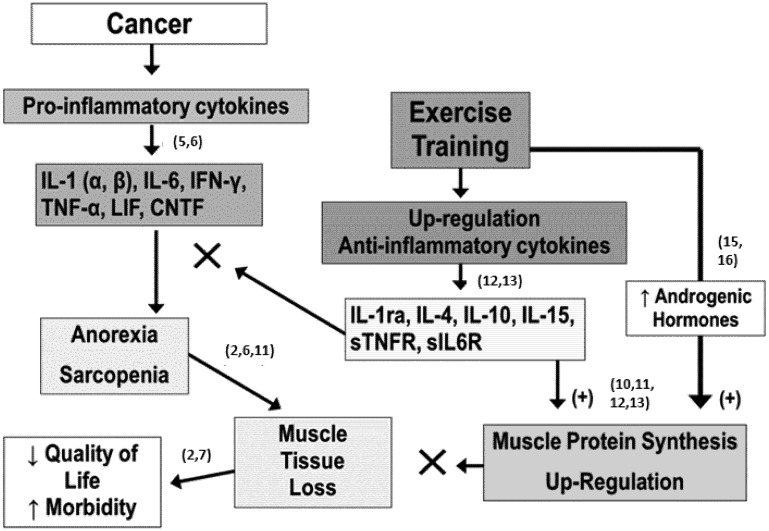
The Exercise Anti-Cachectic Hypothetical (“EACH”) model of the role exercise training has to facilitate the maintenance or accretion of muscle mass in cancer patients and mitigate the effect of anorexigenic pro-inflammatory cytokines. Numbers in brackets refer to reference numbers.

Our hypothetic model of how physical exercise promotes such occurrences is schematically depicted in Figure 1. Cancer and some of the treatments associated with cancer can promote an up-regulation and expression of pro-inflammatory cytokines (see reference [12] for an overview of cytokines’ roles). Many of these cytokines are anorexigenic and/or proteolytic in their actions which promotes behaviors as well as a metabolic state that results in muscle tissue losses (*i.e.*, sarcopenia-like as referred to by other) [3,12]; if unchecked, these physiological responses-adaptations lead to a gradual reduction in functionality and thus quality of life and ultimately a loss of healthy status in the patient. Conversely, regular physical exercise of an appropriate amount is known to promote up-regulation and expression of many anti-inflammatory cytokines and down-regulation of the expression of some detrimental pro-inflammatory cytokines [13,14]. Our model proposes that incorporation of exercise training as a part of the therapeutic regime of cancer treatment can result in these anti-inflammatory cytokines mitigating some of the effects of the pro-inflammatory and through increased androgenic hormonal actions (N.B., exercise enhances the level of such neuro-endocrine agents [15,16]) skeletal muscle protein synthesis is enhanced leading to a reduction in the muscle tissue and net protein losses within the cancer patient.

To date our preliminary findings, both published and unpublished (cytokine changes; ↑ IL-4, IL-10; ↓ TNF-alpha, IL-6) are supportive of this hypothetical model [8,9] as we have shown evidence of improved muscle tissue status (↑ lean body mass) and functionality (↑ strength) in our exercising patients. Furthermore, our initial evidence also supports a shift in the inflammatory cytokine profile of our patients to be more reflective of a healthier status (↑ anti-, ↓ pro-inflammatory cytokines) and an increased proteogenic balance relative to protein turnover (*i.e.*, ↑ positive dietary nitrogen retention; ↑ overall body mass).

It is important to note that due to the myriad of complications that cancer patients can manifest, the approach to an exercise training program in these patients needs to be calibrated on an individualized level [14]. Thus, the time course for changes, the magnitude of changes observed and the tolerance of the exercise program varies notably; nonetheless, we have found cardiovascular (aerobic) and resistance (strength) exercise activities are accepted extremely well and enjoyed when the programs are tailored specifically to the individual patient (as well as monitored closely).

We admit that these findings, by us as well as others, are limited by the relatively few number of exercise studies in cancer patients conducted to date and the low statistical power there within these studies. Nonetheless, the findings are promising and at this stage suggest much further work is needed and warranted in this relatively new and exciting area of integrative physiology involving exercise physiology and oncology medical research.

## 2. Conclusions

We encourage the research practitioners in the field of oncology and all those working in therapeutic-support of cancer patients to examine our Exercise Anti-Cachectic Hypothetical (“EACH”) model (Figure 1), to test it, refine and ultimately improve upon it as a means for us all to strive to a better understanding of how we can aid and help improve the care and lives of cancer patients.
